# Current and Emerging Pharmacotherapeutic Interventions for the Treatment of Peripheral Nerve Disorders

**DOI:** 10.3390/ph15050607

**Published:** 2022-05-15

**Authors:** Jeremy Chung Bo Chiang, Ria Arnold, Roshan Dhanapalaratnam, Maria Markoulli, Arun V. Krishnan

**Affiliations:** 1 School of Optometry and Vision Science, Faculty of Medicine and Health, University of New South Wales, Sydney 2052, Australia; jeremychungbo.chiang@unsw.edu.au (J.C.B.C.); m.markoulli@unsw.edu.au (M.M.); 2 School of Medical, Indigenous and Health Science, Faculty of Science, Medicine and Health, University of Wollongong, Wollongong 2500, Australia; rarnold@uow.edu.au; 3 School of Health Sciences, Faculty of Medicine and Health, University of New South Wales, Sydney 2052, Australia; 4 Prince of Wales Clinical School, University of New South Wales, Sydney 2052, Australia; roshan.dhanapalaratnam@health.nsw.gov.au

**Keywords:** cancer, chemotherapy, chronic kidney failure, diabetes mellitus, immune system diseases, neuropathy, neurotoxicity, obesity, peripheral nervous system diseases, therapeutics

## Abstract

Peripheral nerve disorders are caused by a range of different aetiologies. The range of causes include metabolic conditions such as diabetes, obesity and chronic kidney disease. Diabetic neuropathy may be associated with severe weakness and the loss of sensation, leading to gangrene and amputation in advanced cases. Recent studies have indicated a high prevalence of neuropathy in patients with chronic kidney disease, also known as uraemic neuropathy. Immune-mediated neuropathies including Guillain-Barré syndrome and chronic inflammatory demyelinating polyradiculoneuropathy may cause significant physical disability. As survival rates continue to improve in cancer, the prevalence of treatment complications, such as chemotherapy-induced peripheral neuropathy, has also increased in treated patients and survivors. Notably, peripheral neuropathy associated with these conditions may be chronic and long-lasting, drastically affecting the quality of life of affected individuals, and leading to a large socioeconomic burden. This review article explores some of the major emerging clinical and experimental therapeutic agents that have been investigated for the treatment of peripheral neuropathy due to metabolic, toxic and immune aetiologies.

## 1. Introduction

Peripheral neuropathy is a widespread and debilitating condition that drastically impacts the quality of life of affected patients. Disorders of the peripheral nervous system are often associated with systemic diseases including diabetes, chronic kidney disease and cancer. Large epidemiological studies have been conducted, which show the vast prevalence and impact of the underlying diseases which contribute to peripheral neuropathy. According to the International Diabetes Foundation, the global prevalence of diabetes has been estimated to be 10.5% in 2021, which constitutes approximately 536.6 million people, and this is projected to increase to 12.2% in 2045 [1]. Similarly, chronic kidney disease has a high global prevalence, with an estimated 9.1% of the world’s population being affected in 2017 [2]. The prevalence of peripheral neuropathy is notably high in these diseases, with up to 50% of patients with diabetes affected [3,4]. Cancer is also a global health problem, with 19.3 million new cases diagnosed worldwide in 2020 alone [5]. Neurotoxic chemotherapy is one of the cornerstones of anticancer treatments, with chemotherapy-induced peripheral neuropathy (CIPN) being one of the most common dose-limiting factors, affecting up to 89.4% of treated patients depending on the regimen used [6,7]. As neurotoxic chemotherapy remains a mainstay treatment for some of the most common cancers including breast and colorectal cancers, the prevalence of CIPN is expected to rise [8,9]. Immune-mediated neuropathies are potentially life-threatening conditions, with a mortality rate of up to 13% and a 6.6-fold increased mortality for Guillain-Barré syndrome (GBS) when compared with the background population of the same age [10]. While the prevalence of immune-mediated peripheral neuropathies is approximately 1.90 to 2.81 per 100,000 of the total population [11,12], the underlying conditions have a devastating impact on affected individuals.

Epidemiological evidence has also highlighted the adverse impact of peripheral neuropathy on the lives of affected individuals. In a prospective cohort study (*n* = 7116), peripheral neuropathy was significantly associated with all-cause and cardiovascular mortality, particularly in participants with diabetes [13]. This is of particular concern in older individuals, as demonstrated in a study of elderly people from practices of family physician clinics (*n* = 795), where the prevalence of at least one bilateral sensory deficit including numbness and pain increased from 26% for patients of 65–75 years, to 54% for those aged ≥85 [14]. This association between peripheral neuropathy and age was also reflected in a study with middle-aged (40–69) and older (>70 years) adults from two cohorts in the United States (*n* = 8562) [15]. Sarcopenia is also associated with metabolic conditions, being involved in up to 10.6% of patients with diabetes [16] and is known to be a marker of frailty [17] along with malnutrition [18]. These may further exacerbate the course of peripheral neuropathy, especially in elderly individuals. In terms of psychological impact, breast cancer survivors (*n* = 296) with CIPN up to a mean of 5.6 years since the final chemotherapy treatment have reported greater insomnia severity, anxiety and depression when compared to those without [19]. The same study also showed that higher rates of falls are associated with more severe CIPN [19], which emphasises the functional and physical impact of peripheral neuropathy on individuals.

Due to its severe clinical impact and loss of function, peripheral neuropathy causes significant physical, psychological and economic burden [20,21]. With advancements in diagnostics and improvements in the understanding of underlying pathophysiological mechanisms, there has been an increase in the development of potential pharmacotherapeutic interventions. Given the high global impact of these diseases, this review primarily focuses on the clinical and experimental pharmaceuticals for peripheral neuropathy due to three major aetiologies, namely metabolic disease, immune aetiologies and treatment with neurotoxic chemotherapeutic drugs.

## 2. Metabolic Peripheral Neuropathy

Peripheral nerve injury may occur as a complication of conditions across the spectrum of non-communicable metabolic diseases, including diabetes, chronic kidney disease (CKD) and, as has been more recently been recognised, obesity and the metabolic syndrome [22,23]. Though each of these conditions have unique pathophysiological aetiologies, a common thread is chronic, systemic exposure to altered serum homeostasis. The delicate structure and function of peripheral nerves, especially sensory neurons, are particularly vulnerable to metabolic changes induced by these systemic disorders.

Peripheral neuropathy due to these conditions presents as a distal, symmetric polyneuropathy [24,25]. The length-dependent nature of peripheral neuropathy is demonstrated by direct axonal injury and their symmetrical retraction, or ‘dying back’, with relative preservation of the cell body. Symptoms typically include numbness, tingling, pain and, in advanced cases, unsteadiness or weakness [3]. Recent studies undertaken in cohorts of subjects with diabetes and CKD indicate that about 50% of people will be affected by peripheral neuropathy in their lifetime [3,4]. Despite the staggering impact peripheral neuropathy has on quality of life and health expenditure, the pathophysiology is complex, and disease-modifying treatments remain elusive. Whilst researchers continue to explore promising mechanistic targets and await effective pharmacological interventions, an emerging emphasis on lifestyle interventions and preventative measures provides interim hope for reducing the impact of peripheral neuropathy in these conditions. Hence, this review focuses on these emerging interventions while addressing the pathophysiology of the underlying metabolic peripheral neuropathy.

### 2.1. Pathophysiology Underlying Metabolic Peripheral Neuropathy

**Figure 1:**
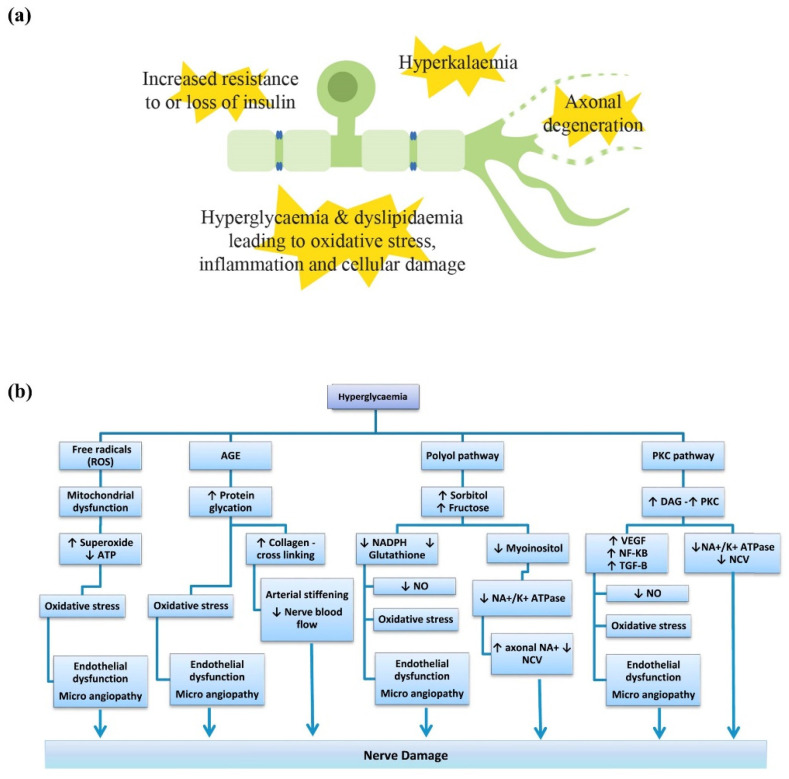
Summary of the pathophysiological mechanisms underlying metabolic peripheral neuropathy. (**a**) For diabetic peripheral neuropathy, these include hyperglycaemic effects, insulin loss or an increased resistance of neurons to insulin, normally required as a neurotrophic factor, which leads to inflammation, production of reactive oxygen species and cellular damage. In chronic kidney disease, hyperkalaemia has also been implicated, regardless of diabetic or non-diabetic aetiology. Emerging evidence also points towards axonal degeneration induced by sterile alpha- and T1R-motif-containing 1 (SARM1) activation. (**b**) Summary of the pathophysiological pathways leading to nerve damage. Abbreviations: ATP, adenosine triphosphate; AGE, advanced glycation end products; DAG, diacylglycerol; Na^+^/K^+^ ATPase, sodium potassium adenosine triphosphatase; NADPH, nicotinamide adenine dinucleotide phosphate; NCV, nerve conduction velocity; NF-κB, nuclear factor kappa B; NO, nitric oxide; PKC, protein kinase C; TGF-B, transforming growth factor B; VEGF, vascular endothelial growth factor. (**b**) was reprinted from Markoulli et al., and modified for spelling with permission from Elsevier [29].

The contributors to cellular injury in metabolic diseases are numerous and have been summarised in Figure 1. In the context of both Type 1 (T1DM) and Type 2 diabetes (T2DM), hyperglycaemia is a significant contributor to neuronal injury. However, the resistance of peripheral neuropathy to strict glycaemic control alone in T2DM has led to a new focus on other factors that may be present in metabolic syndrome (MetS), such as inflammation and dyslipidaemia, for consideration as potential therapeutic avenues. Insulin signalling remains an important factor with divergent effects in T1DM and T2DM. There have been recent advances in our understanding of neuropathy in chronic kidney disease (CKD) and it has been postulated that long-term potassium dysregulation may have a causal effect in the development of this condition.

#### 2.1.1. Hyperglycaemia

Hyperglycaemia leads to cellular damage in multiple ways. Intracellular glucose in peripheral nerves is resolved largely by glycolysis [26] which, when in excess, causes an accumulation of pyruvate. Pyruvate can then overload mitochondrial processes, including the tricarboxylic acid (TCA) cycle, causing the production of reactive oxygen species (ROS) and the shunting of the metabolism to the lactate pathway, eventually depleting nicotinamide adenine dinucleotide (NAD^+^) and leading to the inhibition of glycolysis, further exacerbating neuronal dysfunction [26]. When glycolysis is overwhelmed, other pathways are activated, including the polyol pathway and the hexosamine pathway [26]. The polyol pathway utilises aldose reductase to reduce glucose to sorbitol, which is then converted to fructose by sorbitol dehydrogenase, utilising NAD and consuming the cofactor nicotinamide adenine dinucleotide phosphate (NADPH). This activity can alter the ratio of reduced NAD to oxidised NAD causing osmotic stress, which in turn further increases the production of ROS. The accumulation of sorbitol and fructose also leads to a reduction in myoinositol needed for normal nerve function, which subsequently decreases membrane sodium potassium adenosine triphosphatase activity (Na^+^/K^+^ ATPase) and causes nerve dysfunction [27]. The protein kinase C (PKC) could also be activated by diacylglycerol (DAG) in a high glucose environment [28], which further leads to the dysregulation of nerve regeneration and conduction (Figure 1b; reprinted from Markoulli et al., with permission from Elsevier [29]). The hexosamine pathway may also be activated in the setting of sustained substrate overload, which causes neuronal injury via inflammation and mitochondrial toxicity. These events lead to endoplasmic reticulum (ER) stress, DNA damage and ultimately apoptosis [30]. The imbalance in NAD is also thought to lead to axonal degeneration, mediated by the activation of sterile alpha- and T1R-motif-containing 1 (SARM1), which will also be discussed further in the context of CIPN [31].

#### 2.1.2. Dyslipidaemia

In contrast to T1DM, strict glucose control for T2DM only marginally affects peripheral neuropathy outcomes [32], calling into question the only widely accepted disease-modifying treatment for neuropathy in T2DM, namely glycaemic control. Molecular investigations of dyslipidaemia have revealed the influence of various lipids on healthy neuronal function [30,33].

Substrate overload in response to dyslipidaemia in diabetes results in the activation of several pathways, leading to cellular damage [34]. The catabolism of excess non-esterified fatty acids (NEFA) results in the accumulation of acetyl-CoA, feeding into an already strained tricarboxylic acid (TCA) cycle, as well as generating toxic deoxysphingolipids [30]. The accumulation of low-density lipoprotein (LDL) activates a cascade of signalling through a range of receptors including toll-like receptor 4 and receptors for advanced glycation end products (AGE) to induce ROS generation [35]. These proinflammatory signals perpetuate inflammatory ER stress and DNA damage. The convergence of hyperglycaemic and dyslipidaemia cellular pathways to mitochondrial damage and ER stress may represent promising targets for therapeutic intervention.

#### 2.1.3. Insulin

The role of insulin in neuronal function has been dwarfed by the heightened emphasis on its role in glucose uptake, though its role as a potent neurotrophic factor has been well described [36]. The contribution of insulin to the pathogenesis of peripheral neuropathy has recently been investigated, with an emphasis on differing or divergent exposures in T1DM and T2DM. Insulin receptors are widely expressed in sensory and motor neurons structures, with a high density at Schwann cell membranes and the nodes of Ranvier [37]. In T1DM, the loss of insulin is thought to decrease gene expression of essential proteins and protein synthesis, causing cellular injury and initiating apoptosis [38]. The concomitant loss of C-peptide in T1DM appears to exacerbate neuronal injury, and a recent clinical trial has demonstrated improvement with subcutaneous administration of long-acting C-peptide [39]. In T2DM, where early disease causes insulin resistance and chronically elevated insulin levels, the neuropathology diverges from T1DM. The hypothesis that neurons, like other tissues in diabetes, develop insulin resistance has also been postulated [40], although little is known about insulin signalling in the peripheral nervous system, making mechanistic investigations difficult. Some evidence suggests that neurons lose the neurotrophic effect of insulin with chronic exposure, which is possibly mediated by the down-regulation of its receptor or the growth factor pAkt [38].

#### 2.1.4. Potassium

Diabetes is now the leading cause of CKD worldwide [41] and recent studies have demonstrated that diabetic patients with CKD have more severe neuropathy than patients who have either condition alone [42,43]. A large body of work undertaken in patients with CKD, both of diabetic and non-diabetic aetiologies, has demonstrated a strong correlation between hyperkalaemia and the development of both functional and structural changes in peripheral nerves [44,45,46]. A recent randomised clinical trial of potassium restriction in patients with CKD demonstrated that dietary potassium restriction, in conjunction with intermittent sodium resonium administration, was neuroprotective at the 24-month follow-up visit [47]. These data need to be further substantiated in larger trials, with a focus on understanding whether the benefits of potassium restriction vary according to the aetiology of CKD (i.e., diabetic or non-diabetic).

### 2.2. Current Therapies for Metabolic Peripheral Neuropathy

There are currently no approved disease-modifying pharmacotherapeutic treatments for peripheral neuropathy in metabolic disease. Optimal glycaemic control is a mainstay of diabetes care, irrespective of its effect on neuropathy, and has shown a robust effect on peripheral neuropathy in individuals with T1DM but not T2DM [32]. For those with severe neuropathic pain, symptom management may be partially achieved using various medications. Alleviation of pain by 30 to 50% is considered clinically meaningful and may offer an improvement in quality of life [48]. First-line treatments include selective noradrenaline reuptake inhibitors, gabapentinoids and tricyclic antidepressants. Duloxetine, which is a serotonin and noradrenaline reuptake inhibitor, has been shown to have moderate efficacy against diabetic peripheral neuropathy [49]. However, these medications mainly address symptoms, without any definite impact on neuropathy progression. Opioids have shown efficacy but should be avoided given the evidence demonstrating adverse outcomes such as potential dependence and overdose [30]. It is important to note that few patients experience a complete resolution of their painful symptoms, and symptom management with currently approved medications does not influence the root cause or progression of peripheral neuropathy. While aldose reductase inhibitors were also proposed for the treatment of diabetic peripheral neuropathy by reducing glucose flux, equivocal findings from preclinical studies and insufficient evidence for its efficacy in alleviating signs of symptoms in clinical studies suggests that other mechanisms may play a larger role in the pathogenesis of peripheral neuropathy [50,51,52].

### 2.3. Emerging Pharmacotherapeutics in Metabolic Peripheral Neuropathy

#### 2.3.1. Glucagon-like Peptide-1 (GLP-1) Receptor Agonists

GLP-1 receptor agonists are an established treatment for Type 2 diabetes management, with benefits for lowering blood glucose and often inducing weight loss. This is achieved through augmentation of insulin responsiveness and a reduction in appetite [53]. A wealth of animal studies using GLP-1 receptor agonists have demonstrated improvements in nerve function [54,55,56,57,58,59,60,61]. Importantly, GLP-1 receptors are known to also be expressed in the peripheral nervous system [62] and their mechanism of action is postulated to be through direct effect on neural pathways that influence insulin signalling, increase microvascular perfusion and heighten nerve sodium–potassium pump function, shown in both preclinical and clinical studies [56,59,63,64], which directly address some of the pathophysiological pathways described earlier. These actions ultimately reduce oxidative stress and modulate inflammatory pathways that contribute to neurodegeneration. A recent clinical study of exenatide demonstrated improvements in nerve function that were noted in both cross-sectional and prospective neurophysiological studies [64]. While duloxetine has been shown to be partially effective in alleviating symptoms in clinical studies for diabetic peripheral neuropathy in the short term [49], evidence of its acute and long-term impact on nerve function and morphology, which are indicative of peripheral nerve health, remain limited from a preclinical and clinical perspective. This contrasts with exenatide, which has been shown to lead to the regeneration of myelinated nerve fibres and improvements in nerve conduction parameters in animal studies [59].

Despite these encouraging findings, an 18-month randomised controlled trial of exenatide demonstrated no significant effect on neuropathy measures including symptomatology and intraepidermal nerve fibre density in participants with mild to moderate peripheral neuropathy when compared to insulin treatment, a mainstay of glycaemic care [65]. The investigators suggested that this result could be related to the stage of neuropathy, which may have been too advanced to be amenable to treatment [65]. As such, further investigation with more sensitive measures and targeted trial recruitment is warranted.

#### 2.3.2. Lifestyle and Exercise Interventions

Exercise and physical activity interventions provide hope for improving neuropathy status, glycemia and lipid profile, inflammatory status, physical ability and possibly pain tolerance [66]. While health promotion is a key strategy in clinical management of metabolic disease and arguably already embedded in routine clinical care [67], global trends for physical activity suggest these efforts are not having the desired effect [68]. When strictly assessing the literature evaluating neuropathy endpoints, rather than physical performance endpoints, the evidence is preliminary but promising. A randomised controlled trial demonstrated that exercise improved some nerve conduction parameters and vibration perception and, importantly, significantly reduced the development of neuropathy over the 4 year duration of the study [69]. While this study has several limitations including the small sample size, it was the first to demonstrate that long-term exercise training had preventative effects on the development of peripheral neuropathy when compared to antidiabetic drug regimens alone [69]. Since then, another randomised trial in patients with diabetic neuropathy has demonstrated the efficacy of exercise on various measures, including the Michigan Neuropathy Screening Instrument scores and vibration perception threshold, in addition to unspecified antidiabetic medications [70]. Other uncontrolled studies using an intensive exercise programme have shown positive neuropathy outcomes including an improved intraepidermal nerve growth on skin biopsy in cohorts with established diabetes [71,72]. In the context of prediabetes, studies of epidermal regeneration have shown a promising enhancement with exercise intervention, alongside reductions in pain [73,74]. While these results are encouraging, few studies have included large sample sizes and control groups. Further well-designed, long-term studies measuring gold standard neuropathy outcomes are needed to strengthen the evidence base, for which the results of the recently commenced ADAPT trial may provide important insights [75]. Additionally, cost–benefit analyses are needed for ongoing allied health professionals in order to promote the adoption, adherence and maintenance of lifestyle interventions, and to inform their suitability for routine clinical translation [76].

#### 2.3.3. Bariatric Surgery and Medical Weight Loss

Historically, there have been concerns regarding the development of neuropathy as a complication of bariatric surgery. However, recent evidence suggests that bariatric surgery has a positive effect on peripheral neuropathy [77]. Further encouraging evidence for improvements in microvascular complications have been noted in a large retrospective, matched-cohort analysis, which demonstrated a lower cumulative risk of peripheral neuropathy over approximately 4 years in 4024 people who underwent bariatric surgery, compared to 11,059 who were managed non-surgically [78]. Given the recent change in the focus on mechanisms causing neuropathy, there has been great interest to determine the impact of weight loss strategies on the development of neuropathy in people with severe obesity. Two recent observational studies, one in an exclusively non-diabetic group [79] and the other in a cohort of patients with diabetes [80] who were followed for 12 months after surgery, demonstrated improvements in small nerve fibre parameters and symptom scores. However, in both studies, measures of large fibre function and nerve conduction did not improve [79,80]. Another prospective study of participants attending a medical weight management program showed an improvement in symptomology and a stability of intraepidermal nerve fibre density over 2 years [81]. Taken together, these findings suggest that improvements in metabolic profiles, as may occur in the setting of bariatric surgery or caloric restriction-induced weight loss, may be associated with improvements in small fibre measures and neuropathy symptom scores. Whether physical exercise, as described in Section 2.3.2, has additional beneficial impacts on top of dietary restrictions for peripheral neuropathic measures remains a contentious issue and warrants further investigation [66].

#### 2.3.4. Targeting MicroRNA (miRNA)

MicroRNAs (miRNAs) are small, single-strand, non-coding RNA molecules which regulate various biological processes by interacting with target messenger RNAs or by inhibiting gene translation [82]. Given the improved sophistication in detection methods and the stability of miRNAs, these molecules have been increasingly shown to be involved in a range of diseases, including diabetic peripheral neuropathy. Their up- or down-regulation has been used as a biomarker for neuropathy presence due to their stability in serum as circulating miRNAs, and have been shown to be associated with underlying pathophysiological mechanisms in diabetic peripheral nerve damage, including hyperglycaemia, dyslipidaemia and inflammation [83]. In a genome-wide screening of 1152 miRNAs associated with peripheral sensory neurons in a preclinical mouse model of Type 1 diabetes, miR-380 overexpression was shown to exacerbate diabetes-induced mechanical hypersensitivity and miR-124-potentiated basal mechanical hypersensitivity, while miR-33 overexpression delayed such a hypersensitivity following diabetes induction [84]. In a similar rodent model of streptozotocin-induced diabetes, 49 differentially expressed miRNAs were also found in the Schwann cells of rats with diabetes [83].

Through the use of miRNA mimics or anti-miRs, the biological significance of various miRNAs can be investigated and present potentially vast opportunities for therapeutic targeting. One of the most extensively studied miRNAs in the context of diabetic peripheral neuropathy is miR-146a, which has been shown to be down-regulated in diabetes and promotes a subsequently decreasing distal axonal growth [85], as well as promoting increased inflammation in the sciatic nerve tissues [86]. In a preclinical study on diabetic mice, miR-146a mimics improved nerve conduction velocities, intraepidermal nerve fibre densities, axonal myelination and down-regulated macrophage inflammation in splenic tissues [87]. Other studies using bioengineering innovations such as exosomes enriched with miR-146a [88] or nanoparticle-miR-146a-5p polyplexes [89], in an effort to improve their biocompatibility and safety in future potential clinical applications, have reflected similar positive findings in improving neural function and morphology. Transfecting exosomes, which are membranous nanovesicles that are essential for intercellular transportation and communication, isolated from healthy Schwann cells [90] or mesenchymal stromal cells [91] and placed into diabetic mice have also ameliorated peripheral neuropathy.

While other miRNA mimics [92,93] and anti-miRs [94] that could alleviate diabetic neuropathic pain continue to be discovered, the optimum miRNA target for this purpose remain unknown. Studies are also limited to preclinical investigations, and while findings seem to be highly promising, the clinical efficacy and safety of targeting miRNAs in human studies, compared to other, more established diabetic peripheral neuropathy interventions, will require further investigation.

## 3. Immune-Mediated Peripheral Neuropathy

Immune-mediated neuropathies are a group of peripheral nerve disorders resulting from immune system dysregulation. Patients with these conditions present with sensory disturbance, weakness and ataxia. The aberrant immune response may target either the myelin sheath that surrounds the axonal membrane or the axon itself. From a clinical perspective, distinct forms of immune-mediated neuropathies can be distinguished by their temporal onset, natural history and the involvement of different fibre types, with certain forms targeting motor or sensory fibres and others involving both fibre types. GBS is an acute immune-mediated neuropathy in which people experience a rapid onset of motor and/or sensory deficits over days to weeks, with more severe cases affecting respiratory and autonomic function. There are a number of discrete pathologies that have been associated with GBS including acute inflammatory demyelinating neuropathy, acute motor axonal neuropathy, acute motor and sensory axonal neuropathy and Miller Fisher syndrome [95]. Chronic, immune-mediated neuropathies generally have a duration being greater than 2 months and are defined by their distinct clinical phenotype. These conditions include a number of distinct clinical disease states, namely chronic inflammatory demyelinating polyneuropathy (CIDP), multifocal motor neuropathy (MMN), multifocal acquired demyelinating sensory and motor neuropathy, chronic immune sensory neuropathy, distal acquired demyelinating symmetric neuropathy, demyelinating neuropathy with central nervous system demyelination, gait ataxia with late onset polyneuropathy, chronic ataxic neuropathy with ophthalmoplegia, immunoglobulin M (IgM) paraprotein, cold agglutinins and disialosyl antibodies and immune-mediated neuropathies that are associated with IgM, as well as monoclonal gammopathy of uncertain significance [96].

### 3.1. Pathophysiology of Underlying Immune-Mediated Peripheral Neuropathy

The pathogenesis of GBS is proposed to follow the model of molecular mimicry, typically triggered by an antecedent infection with a similar epitope to those found on peripheral nerve axons [97]. The most common organism to trigger this response in GBS is Campylobacter jejuni, which is associated with the development of antibodies against gangliosides, which are glycosphingolipids that are enriched in cell membrane micro-domains and which play an important role in the modulation of ion channel function [98]. These antibodies may be detected in serum taken from patients with GBS and target an array of gangliosides including GM1, GD1a, GD1b and GlaNac-GD1a [99]. Depending on the location of antibody binding, this can produce the various clinical phenotypes seen in GBS based on individual fibre selectivity, such as the acute motor axonal variant associated with anti-GM1 antibodies, sensory ataxia with anti-GD1b antibodies and Miller Fisher syndrome, associated with the anti-GQ1b antibody that is highly expressed at the paranodal regions of extraocular motor nerves [100]. The pathogenic nature of these antibodies is supported by studies that have demonstrated their presence in serum and sural nerve biopsies taken from patients with GBS and the above clinical phenotypes [101].

**Figure 2:**
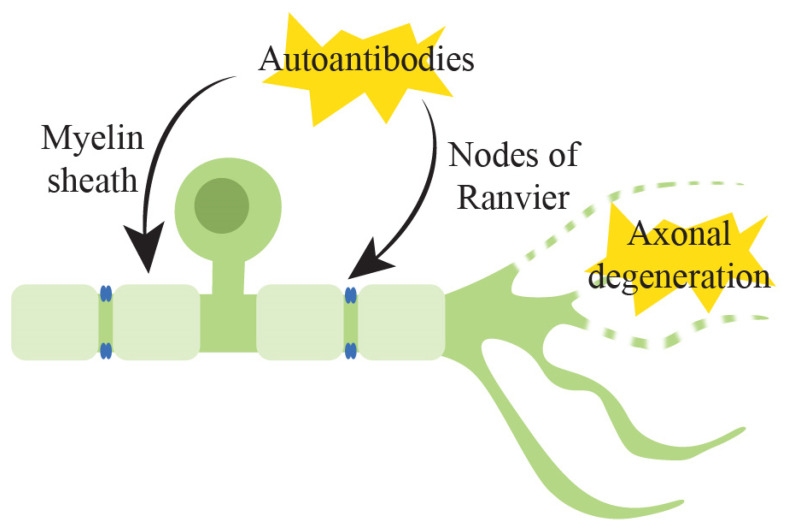
Summary of the pathophysiological mechanisms underlying immune-mediated peripheral neuropathy. These include generation of autoantibodies which bind and cause dysfunction in the proteins or receptors in myelin sheaths, Schwann cells or at the nodes of Ranvier. This may then lead to myelin or axonal destruction.

Most of these conditions involve the generation of autoantibodies, targeting receptors on the myelin sheath or at the nodes of Ranvier, the gap in the myelin sheath between adjacent Schwann cells (Figure 2) [101]. Autoreactive antibodies or immune cells including B and T cells react to glycolipids in Schwann cells, axonal membranes or proteins at the nodes of Ranvier, and antibody-mediated destruction in these regions causes sodium channel dysfunction and paranodal myelin detachment. Binding at this location causes myelin and/or axonal destruction with subsequent damage to the blood–nerve barrier and increased macrophage activity, which then allows plasma proteins to transfer into the cerebrospinal fluid [102]. The destruction of myelin in peripheral nerve fibres leads to a slowing of conduction and, in more severe cases, peripheral nerve conduction failure. In axonal forms of GBS, there is direct injury to the axonal membrane, with antibody- and complement-mediated reactions against axonal membrane epitopes rather than myelin, which causes axolemmal damage and axonal degeneration [101].

The following sections review the major therapies used in clinical settings. Table 1 summarises these treatments and their use in various immune-mediated neuropathies.

### 3.2. Current Therapies for Immune-Mediated Peripheral Neuropathy

#### 3.2.1. Immunoglobulin

Intravenous immunoglobulin (IVIG) has a prominent role in the treatment of immune-mediated neuropathies of all forms. It has become a first-line therapy in GBS and CIDP with evidence for improvement in neurological function [103,104], with a standard induction dosage of 0.4 g/kg, given daily for 5 days. When administered in the therapeutic setting, IVIG is primarily composed of two of the four subclasses, namely IgG1 and 2. IVIG has a half-life of 25 days and is typically administered at 4 week intervals for patients with chronic forms of immune-mediated neuropathy, due to its demonstrated long-term efficacy. The use of IVIG as standard therapy in CIDP was supported by findings of the landmark Immune Globulin Intravenous CIDP Efficacy (ICE) trial of 117 participants which demonstrated both short- and long-term benefits for patients with CIDP, with 3 weekly to 4 weekly maintenance IVIG infusions, demonstrating protection against disease relapse [104]. IVIG has also been demonstrated to have strong evidence for longer term remission in a Japanese study of 49 participants, in which 70% achieved remission for 12 months when IVIG was administered at a dose of 1 g/kg every 3 weeks [105]. A recent clinical trial evaluated different maintenance dosages of IVIG for patients with CIDP, namely 0.5, 1 and 2 g/kg, administered at 3 week intervals. The study demonstrated that a maintenance dose of 2 g/kg when compared to 0.5 g/kg was associated with a better long term disease remission. Both of these dosages did not have a statistical difference to the cohort receiving 1 g/kg of IVIG [106]. IVIG has is also considered standard therapy for the treatment of multifical motor neuropathy (MMN) and leads to improved clinical and electrophysiological outcomes [107,108]. Studies have demonstrated the efficacy of IVIG on muscle strength and in preventing long term disability in MMN [109]. While IVIG is considered a safe form of neurological treatment, rare adverse effects include hypersensitivity reactions, thromboembolism, aseptic meningitis and haemolytic anaemia.

It is proposed that IVIG exerts its benefit by altering the activation threshold of activating and inhibitory constant fragment receptors (FcyRs) such that the immune response can be modified when IgG molecules bind at these sites [110]. It has also been proposed that IVIG may inhibit the production of antibodies, as well as increasing the catabolism of these disease-causing antibodies. Anti-idiotypic antibodies in IVIG are suggested to cause the neutralisation of pathogenic antibodies, and to affect other mechanisms including the modulation of T and B cell expression, cytokine production and the induction of remyelination [111].

More recently, subcutaneous immunoglobulin (SCIG) therapy has been demonstrated to be non-inferior to IVIG for the maintenance therapy of immune-mediated neuropathies such as CIDP [112], and is advantageous in its ease of administration and practicality, as it is able to be self-administered at home. SCIG use has demonstrated a superiority to placebo and is associated with a decreased relapse rate or treatment discontinuation in CIDP [112,113]. In one prospective study, SCIG has demonstrated non-inferiority to IVIG in a cohort of participants who switched from IVIG to SCIG [114].

#### 3.2.2. Corticosteroids

Multiple studies have demonstrated that corticosteroids are effective as a first-line treatment for CIDP [115,116]. Corticosteroids have a half-life of 3 to 4 h and can be given by the intravenous or oral route, with the aim of modulating the protein receptors in cellular cytoplasm to suppress the inflammatory cascade. Intravenous methylprednisolone, given over 3 to 5 days, has been demonstrated to improve motor outcomes in CIDP [117]. Oral courses of corticosteroids of 60 mg per day, tapered over a 3 month period, have also led to improved functional and electrodiagnostic outcomes [118]. Potential adverse reactions that should be noted with the chronic use of corticosteroids include cataracts, hyperglycaemia, weight gain, fluid retention, peptic ulcer disease, osteoporosis, avascular necrosis of the femoral head and opportunistic infection.

#### 3.2.3. Plasma Exchange

Plasma exchange or plasmapheresis has historically been known to be an effective alternate first-line therapy in GBS and has also been shown to be effective in other immune-mediated neuropathies [119]. It is postulated that this treatment filters out autoantibodies, which mediate the inflammatory response. The plasma volume that is removed is typically replaced with autologous filtered plasma, donor plasma or plasma substitute. A randomised trial comparing IVIG and plasma exchange as treatment for GBS demonstrated no significant difference between the two treatment types, with improved neurological outcomes noted in both treatment arms [103]. A study of the utility of plasma exchange for GBS demonstrated that two plasma exchanges undertaken 2 days apart shortened the time to the onset of motor recovery, and that four plasma exchanges on alternate days was superior to this [120]. Current clinical practice has shifted towards a total of five exchanges. Adverse effects are usually associated with the use of citrate (hypocalcaemia or metabolic alkalosis) or haemodynamic or cardiovascular instability. In addition to first-line therapy in GBS, plasmapheresis has shown efficacy for short-term use in CIDP during relapses, with improvements in nerve conduction parameters and neurological function [121].

#### 3.2.4. Cyclophosphamide

Cyclophosphamide has been used for immune-mediated neuropathy cases that are unresponsive to first-line therapy [122]. Cyclophosphamide is an alkylating agent and has an immunosuppressive effect by inhibiting protein synthesis via the cross-linking of DNA and RNA strands. An initial intravenous induction of cyclophosphamide followed by oral maintenance therapy has been demonstrated to be effective in improved motor outcomes in 50% of patients with MMN, and was superior to low-dose oral cyclophosphamide [123,124]. Cyclophosphamide has been shown to promote long periods of clinical remission after monthly administration for 6 months in CIDP [125]. Despite its role in refractory disease, cyclophosphamide should be used with caution due to an increased risk of infertility, teratogenicity, malignancy and haemorrhagic cystitis.

#### 3.2.5. Rituximab

Rituximab is an anti-CD20 mouse–human chimeric antibody with demonstrated evidence in several conditions including chronic immune-mediated neuropathies. It mediates its effects by reducing autoreactive B cells and enabling the production of newer B cells to reestablish tolerance [126]. A study of 26 participants randomised to placebo vs. rituximab for anti-MAG (myelin-associated glycoprotein) neuropathy, a subtype of CIDP, demonstrated an increased rate of improvement in the neuropathy scores of four participants in the treatment group vs. none in the placebo group, which was found to be statistically significant [127]. A more recent, placebo-controlled trial that was undertaken in 54 patients with anti-MAG neuropathy demonstrated that no significant change in the primary outcome was related to neuropathy sensory scores, although significant changes were noted in secondary outcomes, including neuropathy-related disability and quality-of-life scores [128]. With respect to rituximab treatment in MMN, an open-label trial demonstrated no significant change in IVIG usage or neuropathy scores, although no adverse events were noted, which suggested that rituximab can be safely administered to patients with this condition [129]. A case series of three patients with MMN who were treated with rituximab demonstrated sustained clinical benefit and suggested that this may be an acceptable therapy for patients with MMN who become less responsive to IVIG [130]. There is also evidence of its use in other immune-mediated neuropathies such as anti-MAG neuropathy [131], and there is growing evidence for its use in a subgroup of patients with CIDP, in whom serum studies have demonstrated the presence of specific antibodies to neurofascin and contactin, which are transmembrane adhesion molecules located in the paranodal region of peripheral nerves [132].

### 3.3. Emerging Pharmacotherapeutics in Immune-Mediated Peripheral Neuropathy

#### 3.3.1. Eculizumab

Eculizumab is a humanised monoclonal antibody that binds to and blocks cleavage of C5, preventing the cascade of effects of pro-inflammatory C5a and C5b-9. There is early evidence that it has a role in immune-mediated neuropathy, by way of intervening in the complement cascade of the immune response [133]. Eculizumab has been shown to prevent electrophysiological and structural changes in Miller Fisher syndrome for human neuromuscular junctions in vitro and in vivo with mouse models [134]. Eculizumab was shown to prevent respiratory paralysis in these models, providing support for wider human clinical trials. A multicentre, double-blinded, randomised phase two trial has demonstrated its safety and efficacy for GBS treatment [135]. A recent meta-analysis of two clinical trials, including the one mentioned, indicated the likelihood for eculizumab in improving disability grades, however, as only a total of 41 participants have been investigated across both trials, it is clear that larger studies are needed to confirm any clinical benefits of eculizumab [136].

#### 3.3.2. Targeting Antigen-Presenting Cells

Emerging pharmacotherapeutics are aimed at targeting more specific elements of the immune system. While rituximab induces the depletion of B cells and eculizumab targets the complement cascade, other cells involved in the innate immune system which initiate an adaptive response have recently been of interest. Antigen-presenting cells such as dendritic cells and macrophages have also been implicated in immune-mediated peripheral neuropathies. In a B7-2 knockout mice model of spontaneous autoimmune polyneuropathy, which mimics CIDP, an increase of CD11b^+^ dendritic cells were found with an impaired antigen-presenting function due to the deficiency in B7-2, which is also responsible for tolerance induction [137]. However, the adoptive transfer of B7-2 knock-out dendritic cells treated with IL-10 (200 ng/mL) for 3 days in vitro into affected mice successfully led to tolerance and an improvement in clinical scores, grip strength and electrophysiologic parameters [137]. IL-10-preconditioned B7-2-knockout dendritic cells more closely mimicked wild-type dendritic cells and demonstrated an improved ability to generate regulatory T cells, although it did not impact on antigen uptake capabilities [137].

In another experimental study on myelin-homogenate-induced Lewis rat experimental autoimmune neuritis, a model for GBS, quinpramine treatment (2 mg/kg/week) significantly reduced the clinical severity of the neuritis by reducing the infiltration of macrophages into peripheral nerves [138]. On the molecular level, quinpramine, which is a hybrid compound derived from quinacrine and imipramine, reduced peripheral nerve inflammation and demyelination by reducing the cell surface presence of major histocompatibility complex (MHC)-II, subsequently reducing myelin reactive T cells [138]. Recent preclinical studies have also demonstrated that Schwann cells could also act as antigen-presenting cells through the expression of MHC-II and are involved in neuroinflammation [139,140]. Hence, pharmacotherapeutics that can modulate antigen-presenting capabilities may be of growing importance as a treatment strategy for immune-mediated peripheral neuropathies.

## 4. Chemotherapy-Induced Peripheral Neuropathy

Neurotoxicity from cancer chemotherapy is one of the most common dose-limiting factors, with a prevalence of up to 70% within 6 months after chemotherapy treatment [8,9]. Chemotherapy-induced peripheral neuropathy (CIPN) typically presents with symmetrical numbness, paraesthesia or pain in the distal limbs. Notably, there is currently no clinically established treatment, nor any preventive strategies approved for CIPN. This stems from an incomplete understanding of the underlying pathophysiology of the condition. Studies in CIPN investigating the potential to prevent this neurotoxicity from developing provides a unique opportunity for pharmaceutical development, particularly when compared to metabolic neuropathies, where the timing of neuropathy onset is difficult to predict. Early intervention could then be considered in patients who demonstrate subtle changes in nerve function.

### 4.1. Pathophysiology Underlying Neurotoxicity

#### 4.1.1. Neurotoxic Chemotherapy

**Figure 3:**
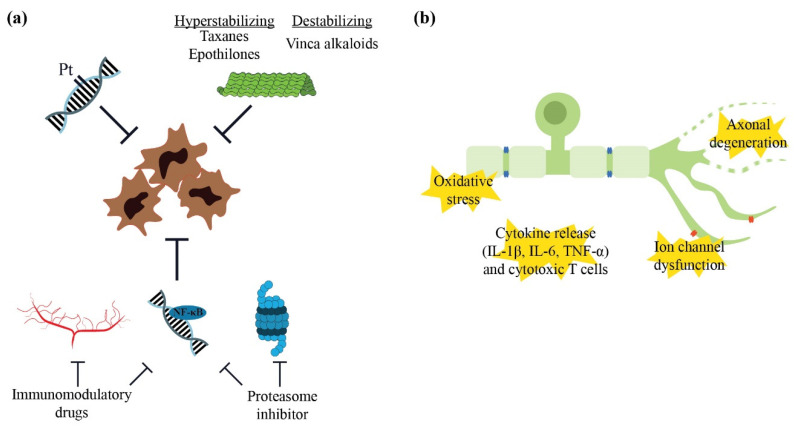
Underlying antitumoral mechanisms and pathophysiology of peripheral neuropathy: (**a**) Antitumoral mechanisms of neurotoxic chemotherapy drugs including the formation of platinum (Pt)-DNA adducts (platinum compounds), antimicrotubule formation (taxanes, epothilones, vinca alkaloids), proteasome inhibition (proteasome inhibitors), antiangiogenic effects and down-regulation of NF-κB (immunomodulatory drugs); (**b**) Additional pathophysiological mechanisms underlying chemotherapy-induced peripheral neuropathy (CIPN). These include oxidative stress with mitochondrial dysfunction, neuroinflammation involving increased cytokine release and cytotoxic T cells and ion channel dysfunction. Other signalling pathways leading to eventual axonal degeneration, including sterile alpha- and T1R-motif-containing 1 (SARM1) activation, are also involved.

Several forms of chemotherapy have been implicated in contributing to neurotoxicity and causing CIPN including platinum compounds, taxanes, vinca alkaloids, epothilones, proteasome inhibitors and immunomodulatory drugs. Each group exerts different cytotoxic mechanisms as summarised in Figure 3. Platinum compounds, including cisplatin and oxaliplatin, form platinum-DNA adducts, preventing further cellular division [141]. Taxanes, vinca alkaloids and epothilones affect microtubule stability and polymerisation, which inhibit cellular proliferation [142,143]. Proteasome inhibitors such as bortezomib, inhibit proteasome, a large complex protease that is responsible for proper signal transduction and stress response by identifying and degrading proteins. Dysregulation of this system triggers cell cycle arrest and apoptosis [144]. The anti-inflammatory and anti-angiogenic effects of immunomodulatory drugs, namely thalidomide, also contribute to combat cancer growth. Both bortezomib and thalidomide have been shown to block or reduce NF-κB activity, which is involved in DNA transcription and cytokine production that mediate cellular growth [143,145]. However, the antineoplastic mechanisms of these chemotherapeutic drugs also impact on neuronal elements which lead to neurological symptoms.

#### 4.1.2. Mechanisms of Neurotoxicity

Numerous mechanisms have been identified which contribute to or lead to CIPN, mainly through animal or in vitro studies (Figure 3). Mitochondrial dysfunction from oxidative stress in rodent neuronal cell cultures, including those from the dorsal root ganglia, has been demonstrated with platinum compounds [146] and bortezomib [147]. Dysregulation of ROS, which further contributes to oxidative stress, also leads to the hypersensitivity of peripheral nociceptors in mice treated with cisplatin or paclitaxel [148]. Mitochondrial toxicity eventually leads to cellular death and has also been implicated in the impairment of Schwann cells, the main glial cells in the peripheral nervous system which provide neuronal support [149].

Neuroinflammation has recently emerged as a potential contributor to CIPN development and progression. Up-regulation of inflammatory cytokines including interleukin (IL)-1β, IL-6 and tumour necrosis factor (TNF)-α, as well as the overexpression of cytotoxic CD4+ and CD8+ T cells of the adaptive immune system in the dorsal root ganglia and peripheral neurons, have been observed with preclinical models of oxaliplatin [150], paclitaxel [151,152] and vincristine neurotoxicity [153]. Dendritic cells, which are the most potent antigen-presenting cells and initiators of the immune system, have also been shown to be present in elevated numbers in other regions, such as the cornea in patients who were treated with oxaliplatin [154].

The dysregulation of membrane channels has also been implicated in neurotoxicity. Extensive neurophysiological studies in animals and humans have supported the role of the up-regulation of transient voltage-gated sodium ion (Na_v_) channels in the development of peripheral neuropathy from oxaliplatin [155,156,157], paclitaxel [158] and vinca alkaloids [159]. Additionally, the expression of transient receptor potential (TRP) channel family members, which signal noxious mechanical and heat (TRP vanilloid 1; TRPV1) or cold sensations (TRP metastatin 8; TRPM8), have also been shown to be up-regulated in the dorsal root ganglia neurons of rats treated with oxaliplatin [160], paclitaxel [161] or vincristine [162].

Numerous molecular signalling pathways have been identified in recent times, some of which are associated with the above-mentioned pathophysiological mechanisms, including oxidative stress and neuroinflammation. Researchers have steadily unravelled these in an effort to identify potential neuroprotective and antinociceptive targets that may prevent or treat CIPN, rather than depending purely on symptomatic treatments.

### 4.2. Current Therapies for Chemotherapy-Induced Peripheral Neuropathy

In contrast to immune-mediated peripheral neuropathies, there is currently an absence of clinically established pharmacotherapeutic interventions for preventing or treating CIPN. Duloxetine, which is conventionally used as an antidepressant medication and has been mentioned previously in the diabetic peripheral neuropathy section, has also been recommended for alleviating CIPN symptoms [163,164]. Recent preclinical evidence has shown a potential neuroprotective impact of duloxetine by preventing the damage of intraepidermal nerve fibre densities and by reducing the mRNA expression of IL-6 and TNF-α during oxaliplatin or paclitaxel administration in mice [165]. However, clinical studies still show a potential lack of efficacy [166,167], with low tolerability, male gender and chronic CIPN potentially limiting duloxetine use clinical in settings [167].

Clinical pharmaceutical interventions such as opioids and other analgesics [168,169], repurposed drugs such as other SNRI [170], anticonvulsants [171,172], antidiabetic medications [173] and poly (ADP-ribose) polymerase inhibitors [174], as well as dietary supplementation or alternative medicines [175,176,177,178,179] have shown limited evidence for CIPN prevention and treatment. As discussed in the section on metabolic peripheral neuropathies, the use of pain medications primarily addresses symptoms rather than the underlying disease process and this has the potential to be abused, with adverse consequences including addiction or overdosing. The ongoing search for neuroprotective and antinociceptive strategies have revealed more novel targets that could prevent or reverse CIPN particularly in animal studies, including the SARM1 activation of axonal degeneration [31]. However, the translatability of these findings between species and to humans have been equivocal and contentious, further complicating the bench-to-bedside process [180,181]. Instead of focusing on current non-pharmacotherapeutic and therapeutic approaches that have had limited success, such as exercise, conventional analgesics, dietary supplements and drug repositioning efforts, which have been recently reviewed [182,183,184,185,186], the following Section 4.3 will explore two novel, emerging pharmacotherapeutics that have been of particular interest in recent years, in relation to CIPN prevention and treatment, with insights from both preclinical and clinical studies.

### 4.3. Emerging Pharmacotherapeutics in Chemotherapy-Induce Peripheral Neuropathy

#### 4.3.1. Sterile Alpha- and T1R-Motif-Containing 1 (SARM1) and Nicotinamide Adenine Dinucleotide (NAD^+^) Salvage Pathway

##### SARM1 Deletion and Inhibition

**Figure 4:**
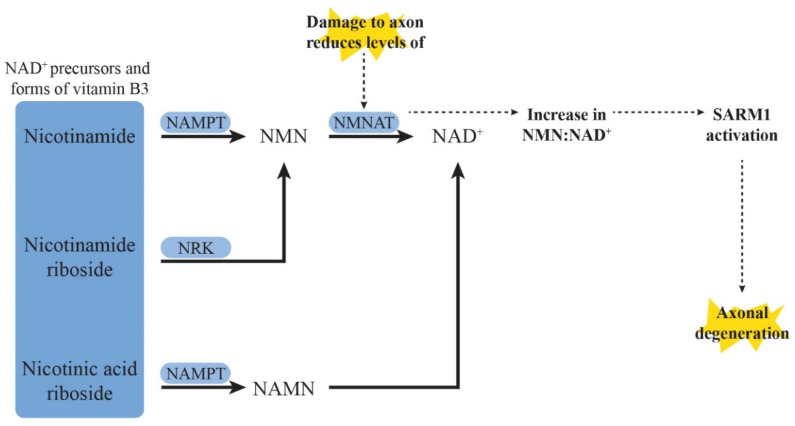
Axonal degenerative mechanisms via the nicotinamide adenine dinucleotide (NAD^+^) signalling pathway and sterile alpha- and T1R-motif containing 1 (SARM1) activation. Damage or insults to axons decreases the availability of nicotinamide mononucleotide adenyltransferase 2 (NMNAT2) at terminal nerve endings due to decreased axonal transport and high turnover rates. The increase in nicotinamide mononucleotide (NMN) and/or decrease in NAD^+^ triggers SARM1 activation, the executor of axonal degeneration through downstream pathways including calcium influx and the mitogen-activated kinase (MAPK) pathways. Other precursors of NAD^+^ or forms of vitamin B3 have also been implicated including nicotinamide riboside (metabolised into NMN by nicotinamide riboside kinase (NRK)) and nicotinic acid riboside (metabolised into nicotinic acid mononucleotide (NAMN) by nicotinamide phosphoribosyltransferase (NAMPT)).

Axonal degeneration is one of the hallmarks of distal sensory peripheral neuropathy and efforts have focused on identifying potential targets of this process for pharmaceutical intervention. SARM1 is a modular protein which has recently been identified as a major mediator of programmed axonal degeneration with its intrinsic NAD-ase enzymatic activity. In healthy neurons, SARM1 is usually inactivated by NAD^+^, which is synthesised from nicotinamide mononucleotide (NMN) by the catalytic protein nicotinamide mononucleotide adenylyltransferase 2 (NMNAT2) (Figure 4) [187]. Neuronal injury or toxicity causes a reduction in NMNAT2 levels due to its rapid turnover, short half-life and the impairment of axonal transport to distal axons [188], leading to an increase in NMN:NAD^+^, which favours the activation of SARM1. While the impact of SARM1 on subsequent pathways is yet to be fully understood, it is thought that its activation leads to calcium influx into the axoplasm of injured axons and the breakdown of neuronal elements [189,190,191].

Most studies involving SARM1 in CIPN have shown promising results at preclinical or experimental stages. In SARM1-knockout mice, the development of typical neuropathic findings such as mechanical and cold hypersensitivity in oxaliplatin [192] and the loss of intraepidermal nerve fibres following paclitaxel treatment were prevented [193,194]. Antagonising cyclic adenosine diphosphate ribose (cADPR), a by-product of SARM1-induced axonal degeneration, with intraperitoneal injection of 8-Br-cADPR, attenuated paclitaxel-induced mechanical hypersensitivity and partially protected against the loss of intraepidermal innervation in mice, without interfering with the antitumoral effects of paclitaxel [191]. This neuroprotective impact is attributed to reduction in axonal calcium flux or inactivation of SARM1. Such a success has also been reflected by the protective effects demonstrated in in vitro studies involving mice and human axons exposed to paclitaxel or vincristine [191,193,195]. Small-molecule isothiazole inhibitors of SARM1 NAD-ase enzymatic activity have been demonstrated to protect against axonal morphological and functional loss in mice treated with paclitaxel [193]. While the findings for electrophysiological studies have been more equivocal, ranging from absence of significant prevention to robust protection with SARM1 deletion, interpretation of the data presented should consider the different dosing strategies and assessment timepoints incorporated in these experimental studies [193,194]. In diabetic peripheral neuropathy, in which axonal degeneration is also a hallmark, SARM1 deletion or deficiency has been shown to alleviate neuropathic impact, including intraepidermal nerve fibre loss, axonal degeneration or growth retardation, and NAD^+^ decrease in streptozotocin-induced diabetic mice [196]. This further elucidates the importance of SARM1 in global axonal degenerative pathways.

##### Targeting the NAD^+^ Signalling Pathway

Several targets in the NAD^+^ signalling pathway that are involved in the upstream modulation of SARM1 expression have also been studied in CIPN models for their potential for restoring the proper balance of NMN and NAD^+^ (Figure 4). Nicotinamide is one of the three forms of vitamin B3 and the principal early precursor of NAD^+^. A study on rats treated with paclitaxel showed that pre-treatment with P7C3-A20 10 mg/kg/day, a stimulator of nicotinamide phosphoribosyltransferase (NAMPT) which converts nicotinamide to NMN needed for NAD^+^ formation, that was administered intraperitoneally prevented intraepidermal nerve fibre degeneration and the development of mechanical and thermal allodynia induced by paclitaxel [197]. Notably, supplementation with nicotinamide fully augmented the neuroprotective efficacy of a subthreshold dose of P7C3-A20 [197]. In another study investigating paclitaxel-induced hypersensitivity in Drosophila larvae, the overexpression of NMNAT, which converts NMN to NAD^+^, partially mitigated thermal hypersensitivity [198].

Nicotinic acid riboside is another precursor of NAD^+^, which is converted to nicotinic acid mononucleotide (NAMN), instead of the pro-axonal degenerative NMN, by NAMPT (Figure 4). In an in vitro model of rat dorsal root ganglion culture treated with vincristine, nicotinic acid riboside and nicotinamide riboside (the third form of vitamin B3 and a precursor of NAD^+^) only conferred partial protection against axonal degeneration [199]. These findings not only imply that repleting NAD^+^ levels may be insufficient in fully restoring neuronal health in neurotoxicity, but in fact may also require the reduction of the heightened levels of NMN which promote axonal degeneration. This is supported by the results of the same study which showed that nicotinic acid riboside that was administered in combination with FK866, an inhibitor of NAMPT, conferred more substantial protection against axonal degeneration by reducing NMN, without compromising NAD^+^ levels [199]. While this seems to conflict with a previously discussed study showing axonal protection with NAMPT stimulators (P7C3-A20) [197], NMN levels were not measured in that study, and it is possible that additional downstream pathways may have been involved, including subsequent NAD^+^ production. More recently, nicotinamide riboside has also been shown to reverse both corneal and hindpaw mechanical hypersensitivity induced by paclitaxel in tumour-bearing rats, although the loss of intraepidermal nerve fibres was not alleviated [200].

While the collective findings of these preclinical studies show that targeting SARM1 and the NAD^+^ pathway seem to be a promising neuroprotective strategy in combatting CIPN, it remains to be seen as to whether future clinical approaches will have similar success. As this strategy is conceptualised from a pathophysiological approach rather than based on symptomatic relief, it may have better success when compared to conventional, repurposed drugs such as duloxetine.

#### 4.3.2. Endocannabinoid System

##### Targeting the Metabolization of Endocannabinoids

The endocannabinoid system in the body is composed of lipid-based ligands, primarily 2-arachidonylglycerol (2-AG) and N-arachidonoylethalomine (AEA), which mainly interact with two presynaptic receptors (cannabinoid receptor 1, CB1 and cannabinoid receptor 2, CB2) to modulate crucial neural responses including pain signalling in both the central and peripheral nervous systems [201,202]. In contrast to the neuroprotective effects of targeting SARM1, stimulating the endocannabinoid system seems to have an antinociceptive effect. Animal studies have revealed a reduction in endocannabinoid levels in the cutaneous distal limb of rodents exposed to neurotoxic chemotherapy drugs including paclitaxel [203] and cisplatin [204], but not in the spinal cord or brain [203,205]. Several studies have also revealed that targeting monoacylglycerol lipase (MAGL), the enzyme responsible for metabolising 2-AG, with synthetic inhibitors such as JZL184 [203,205] and MJN110 [205] alleviated paclitaxel-induced mechanical allodynia in mice. Similarly, the inhibition of fatty acid amide hydrolase (FAAH), which metabolises AEA, has also improved neuropathic pain conditions in rodent models following the administration of paclitaxel [206] or cisplatin [204,207] (Figure 4). Attempts to restore 2-AG and AEA levels through subcutaneous injections of these cannabinoids have shown promising effects in increasing withdrawal thresholds to mechanical stimuli [203,204]. While the antinociceptive action of MAGL inhibitors are thought to be mediated by both CB1 and CB2 interactions [203,205], studies involving FAAH inhibitors have suggested that CB1 is more involved in this process [204,206].

##### Stimulating Endocannabinoid Receptors

Specific agonists of the cannabinoid receptors have also been investigated in treating CIPN. These include CB1 agonists such as the synthetic cannabinoid Hu-210 [208], as well as CB2 agonists, including β-caryophyllene, which is extracted from essential oils of certain plants [209], and the synthetic molecules LY2828360 [210], AM1710 [211] and MDA7 [212], which have shown antiallodynic effects, mostly in models of paclitaxel-induced mechanical hypersensitivity. Notably, some studies have analysed their potential synergy with morphine use, showing that stimulants of the endocannabinoid system could delay morphine tolerance and have synergistic effects with lower doses of morphine or opioid use when administered concomitantly [206,208,211]. These findings may indicate an important role of the endocannabinoid system in pharmacotherapeutic settings to minimise opioid use and dependence for pain control.

In addition to antinociception, stimulating the endocannabinoid system has also attenuated inflammation and dysregulation of ion transporters which are implicated in CIPN pathophysiology. Wu et al., showed a reduction in the pro-inflammatory cytokine IL-6, a rise in in anti-inflammatory IL-10 and the decreased expression of potassium–calcium ion cotransporters when MDA7 was administered in paclitaxel-treated rats [212]. Oral β-caryophyllene also inhibited p38 MAPK/NF-κB activation and IL-1β release in the spinal cord of paclitaxel-treated mice [209], which supports a further downstream impact on inflammation and other pathways in alleviating CIPN.

##### Phytocannabinoids and Cannabis

**Figure 5:**
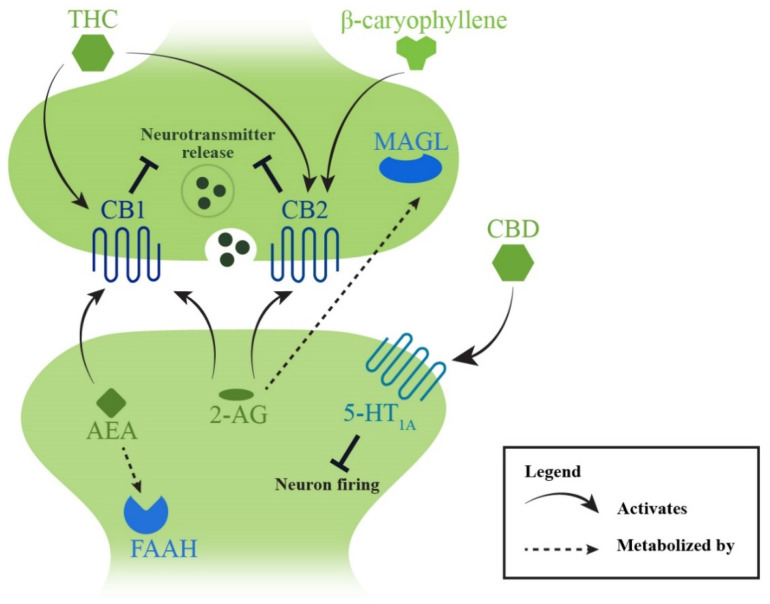
Antinociception via the endocannabinoid system. The two main receptors of this system include cannabinoid receptor 1 (CB1) and cannabinoid receptor 2 (CB2), which are located throughout the central and peripheral nervous systems, with 2-arachidonylglycerol (2-AG) and N-arachidonylethalomine (AEA) as the primary endocannabinoid ligands. Decrease in endocannabinoid levels have been implicated in CIPN pathophysiology, while restoration of the levels and inhibitor of enzymes that metabolise the endocannabinoids have led to antinociceptive effects. Phytocannabinoids derived from cannabis plants (tetrahydrocannabinol (THC) and cannabidiol (CBD)) as well as direct CB1 or CB2 agonists such as β-caryophyllene have also shown similar effects. Abbreviations: 5-HT_1A_, serotonin 1A receptor; FAAH, fatty acid amide hydrolase; MAGL, monoacylglycerol lipase.

Phytocannabinoids are naturally occurring components derived from the cannabis plant, with the two most studied compounds being tetrahydrocannabinol (THC) and cannabidiol (CBD). In studies of paclitaxel-induced peripheral neuropathy, intraperitoneal injections of THC and CBD have shown antiallodynic effects when used by itself and in low dose combinations, with some success also shown in attenuating oxaliplatin- and vincristine-induced mechanical sensitivity [213]. While THC is known to be a non-selective CB1 and CB2 agonist, cannabidiol interacts more so with other receptors, including the serotonin 1A receptor (5-HT_1A_), to alleviate neuropathic pain [214] (Figure 5). A recent study also showed that the protective effect of CBD and KLS-13109 (a CBD analogue) may also be mediated by a mitochondrial sodium–calcium ion exchanger [215].

Human clinical trials have been conducted on the use of cannabis and phytocannabinoids with mixed results. Nabiximol, which is an oral mucosal spray consisting of equal parts of THC and CBD, was shown to be ineffective in treating CIPN, although the study had a small sample size and recruited patients who were treated with a variety of neurotoxic drugs including platinum compounds, paclitaxel and vincristine [216]. A more recent retrospective analysis on patients who were treated with medicinal cannabis prior to or within the first month of commencing oxaliplatin chemotherapy showed that 59.7% were free from neuropathy when compared to 31.7% in the control group, without cannabis treatment [217]. While targeting the endocannabinoid system seems to have some success in animal models, it is not currently recommended for clinical use due to the equivocal findings in human studies and lack of strong evidence. Due to the paucity of evidence for its clinical application in CIPN, it is difficult to compare its antinociceptive potential when compared with drugs currently used particularly duloxetine. It is evident that neural, morphological and electrophysiological studies should be conducted to further evaluate whether targeting the endocannabinoid system for CIPN confers appreciable clinical improvements and to investigate any potential neuroprotective benefits.

## 5. Overall Conclusions

As noted throughout this review, a deep understanding of the pathophysiological mechanisms underlying the peripheral neuropathy associated with various conditions is crucial for developing effective treatments. While pharmacotherapeutic interventions have been more established in clinical settings for the treatment of immune-mediated neuropathies, there has been a notable lack of effective treatment for metabolic peripheral neuropathies and CIPN. The interventions used for metabolic peripheral neuropathies are primarily for symptom management, with limited consideration for the underlying cause of progression of peripheral nerve damage. In terms of CIPN, the potential different mechanistic pathways associated with each drug type further adds to the complexity of finding an effective treatment that can combat CIPN that is caused by the different drug types. More rigorous clinical and experimental studies with standardised methodologies and investigations of specific neurotoxic chemotherapeutic drugs are also required to address this gap to develop more effective therapies. A shift from therapeutics, based primarily on symptomatic management, to those that target specific pathophysiological mechanisms underlying the development or progression of peripheral neuropathy would be desirable in an effort to combat this debilitating condition. In terms of preclinical investigations, while targeting SARM1 has been a major interest in CIPN, which is likely due to its neuroprotective potential, targeting miRNAs have been more of a focus in diabetic peripheral neuropathy in recent years. Limited and sparse evidence has begun to emerge in the field of diabetic peripheral neuropathy and CIPN, which demonstrate the potential for SARM1 or miRNA, respectively, as targets for therapeutic intervention [196,218]. A cross-disciplinary approach may help integrate knowledge gained from different approaches and further promote the clinical application of successful therapeutic interventions. Targeting a combination of multiple mechanisms may also be a potential novel avenue in therapeutic research, as each approach may have a synergistic impact on another in terms of neuroprotection and/or antinociception. In the advent of epigenetics and the discovery of axonal degenerative pathways, more successful, targeted pharmacotherapeutics may be developed in the future.

## Figures and Tables

**Table 1:** Treatments for immune-mediated neuropathies.

Treatment	Disease	Route/Dose	Half-Life	Adverse Effect
Immunoglobulin	GBS CIDP MMN	Intravenous, 0.4 g/kg daily for 5 days or subcutaneous	25 days	hypersensitivity reactions, thromboembolism, aseptic meningitis and haemolytic anaemia
Plasma exchange	GBS CIDP MMN	Intravenous 4–5 exchanges on alternate days	1–2 months	Citrate-related (hypocalcaemia or metabolic alkalosis) or cardiovascular instability
Corticosteroids	CIDP	Intravenous 1 g daily for 3–5 days Oral 60 mg daily, tapered over 3 months	2–4 h	Cataracts, hyperglycaemia, weight gain, fluid retention peptic ulcer disease, osteoporosis, avascular necrosis of the femoral head and opportunistic infection
Cyclophosphamide	CIDP MMN	Intravenous 40–50 mg/kg	6–8 h	Infertility, teratogenicity, malignancy and haemorrhagic cystitis
Rituximab	CIDP MMN	Intravenous 1 g each 2 weeks apart	20.8 days	Neutropenia, leucopenia, opportunistic infection, nausea, rash, fever
Azathioprine	CIDP	Oral, 1–2 mg/kg/day	5 h	Lymphoproliferative disorders, skin cancers, hepatotoxicity, GI discomfort
Methotrexate	CIDP	Oral, 7.5–20 mg weekly	6–7 h	Teratogenic, pulmonary fibrosis, hepatotoxicity, GI discomfort
Mycophenolate Mofetil	CIDP	Oral, 1–3 g/day	18 h	Bone marrow suppression, hepatotoxicity, GI upset, infertility

Abbreviations: Guillain-Barré Syndrome, GBS; chronic inflammatory demyelinating polyneuropathy, CIDP; multifocal motor neuropathy, MMN; gastrointestinal, GI.

## Data Availability

Data sharing not applicable.
